# ﻿A new genus and three new species of Lycoperdaceae (Agaricales) from Southern China revealed by molecular phylogeny and taxonomy

**DOI:** 10.3897/mycokeys.118.153703

**Published:** 2025-06-04

**Authors:** Xin Yang, Songjing Duan, Mei Li, Dongxu Li, Rongcong Yang, Shihui Zhang, Taimin Xu, Wen Li, Hongmin Zhou, Changlin Zhao

**Affiliations:** 1 Yunnan Provincial Key Laboratory for Conservation and Utilization of In-forest Resource, the Key Laboratory of Forest Resources Conservation and Utilization in the Southwest Mountains of China, Ministry of Education, Southwest Forestry University, Kunming 650224, China; 2 College of Forestry, Southwest Forestry University, Kunming 650224, China; 3 Yunnan Tongbiguan Provincial Nature Reserve, Mangshi, 678400, China; 4 College of Agriculture and Life Sciences, Zhaotong University, Zhaotong 657000, China

**Keywords:** Classification, molecular systematics, new taxa, taxonomy, puffballs

## Abstract

Lycoperdaceae (Agaricales, Agaricomycetes) is a taxonomically significant fungal family based on the globally distributed and morphologically defined puffball structures. In the present study, one new genus, *Lycoperdia*, and three new species, viz. *Calvatiaphlebioides*, *Lycoperdiatomentosa*, and *Morganellaminima*, collected from southern China are proposed based on a combination of morphological characteristics and molecular evidence. *Calvatiaphlebioides* is characterized by broadly obpyriform to turbinate basidiomes and globose to subglobose basidiospores with a short pedicel. *Lycoperdiatomentosa* is characterized by pyriform basidiomes, tomentose exoperidium, and globose to subglobose basidiospores with a long pedicel. *Morganellaminima* is characterized by tiny basidiomes (5–8 mm in diameter, 3–7 mm in height), globose to subglobose basidiospores with distinct spines, and a short pedicel (<0.5 μm). Sequences of the internal transcribed spacers (ITS), nuclear large subunit ribosomal RNA (nLSU), and RNA polymerase second largest subunit (rpb2) of the nuclear ribosomal DNA (rDNA) markers of the studied samples were generated, and the phylogenetic analyses were performed with maximum likelihood and Bayesian inference methods. The results showed that our collections were clustered within the family Lycoperdaceae. The phylogenetic tree inferred from the ITS+nLSU sequences revealed that all four new taxa were clustered into the family Lycoperdaceae, in which *Lycoperdiatomentosa* formed a monophyletic lineage. The phylogenetic tree inferred from the ITS+nLSU+*rpb2* sequences revealed that one new taxon, *Calvatiaphlebioides*, was clustered into the genus *Calvatia*, sister to *C.longisetulosa*. The phylogenetic tree inferred from the ITS+nLSU sequences revealed that *Morganellaminima* was clustered into the genus *Morganella*, forming a monophyletic lineage. Full morphological descriptions, illustrations, and phylogenetic analysis results for the new genus and three new species are provided.

## ﻿Introduction

The global One Health approach seeks to better understand and improve the interconnectedness of people, animals, plants, and their environment, and such insight will engender a more sustainable future for life on earth, and fungi are essential to this quest because they affect earth’s ecosystems in a myriad of beneficial and detrimental ways (Zhao et al. 2024; Case et al. 2025). Fungi are among the most diverse groups of organisms on this planet and play a core role in ecosystem processes and functioning (Wei and Dai 2004; Cui et al. 2018; Hyde 2022; Dong et al. 2024a; Zhou et al. 2025). Currently, 19 phyla of fungi are accepted: Aphelidiomycota, Ascomycota, Basidiobolomycota, Basidiomycota, Blastocladiomycota, Calcarisporiellomycota, Chytridiomycota, Entomophthoromycota, Entorrhizomycota, Glomeromycota, Kickxellomycota, Monoblepharomycota, Mortierellomycota, Mucoromycota, Neocallimastigomycota, Olpidiomycota, Rozellomycota, Sanchytriomycota, and Zoopagomycota (Zhao et al. 2023b; Wijayawardene et al. 2024). Fungi secrete a spectacular array of bioactive chemical compounds and enzymes; these have crucial roles in the biosphere, from digesting organic matter and recycling nutrients from dead plant and animal tissues to mediating intimate and mutually beneficial associations with the roots of almost all land plants (Wu et al. 2019; Case et al. 2025), in which most Basidiomycota species act as decomposers and mutualists of plants and animals, which play fundamental ecological roles such as driving carbon cycling in forest soils (Tedersoo et al. 2014; Cui et al. 2019; Yuan et al. 2023b).

Lycoperdaceae F. Berchtold & J. Presl is commonly known as puffballs, and species in the family are characterized by the globose to subglobose basidiomes and mature gleba, releasing a powdery mass of spores passively through the apical opening of the endoperidium (Cunningham 1926b; Kreisel 1962; Krüger et al. 2001). These species are widely distributed in temperate, arid, and tropical climates, found on the forest floor, rotting wood, or open meadows (Pegler et al. 1995). Some species within this family have culinary and medicinal values, such as *Calvatiacraniiformis* (Schwein.) Fr. ex De Toni and *C.cyathiformis* (Bosc) Morgan are used as food in the UK, while *C.gigantea* (Batsch) Lloyd and *C.lilacina* (Mont. & Berk.) Henn are used in traditional medicine for their hemostatic properties and to treat various ailments (Coetzee and van Wyk 2009).

Lycoperdaceae was established by F. Berchtold and J. Presl in 1820, originally encompassed 18 genera and 150 species, and initially this family was considered synonymous with Agaricaceae (Kirk et al. 2008); later, the molecular phylogenetic analyses revealed this family, Lycoperdaceae, to be a monophyletic gasteroid lineage distinct from Agaricaceae (He et al. 2019). Subsequent studies identified four main subclades within Lycoperdaceae as the genera *Bovista* Pers., *Calvatia* Fr., *Disciseda* Czern., and *Lycoperdon* Pers. *sensu lato* (Larsson and Jeppson 2008). In the recent taxonomic system of Basidiomycota, Lycoperdaceae was treated as a distinct family comprising the genera *Apioperdon* (Kreisel & D. Krüger) Vizzini, *Gastropila* Homrich & J.E. Wright, *Bovista*, *Bryoperdon*, *Calvatia*, *Calbovista* Morse ex M.T. Seidl., *Lycoperdon* Pers., and *Morganella* Zeller (He et al. 2019). However, certain puffball-like genera were still accommodated within Agaricaceae, such as *Abstoma*, *Acutocapillitium* P. Ponce de León, *Arachnion* Schwein., *Calvatiopsis* Hollós, *Disciseda* Czern, *Glyptoderma* R. Heim & Perr. – Bertr., *Japonogaster* Kobayasi, and *Lycoperdopsis* Henn. (He et al. 2019). Conversely, a recent compendium of generic names for agarics and Agaricales, Kalichman et al. (2020), proposed the concept of Agaricaceae s.l., in which this group includes five families: Lycoperdaceae, Agaricaceae s. str., Coprinaceae, Lepiotaceae, and Tulostomataceae. Lycoperdaceae has more recently been acknowledged as an independent family (He et al. 2019; Kalichman et al. 2020), even though historically it was treated as synonymous with Agaricaceae (Kirk et al. 2008). Both Index Fungorum (http://www.indexfungorum.org; 23 March 2025) and the MycoBank database (http://www.MycoBank.org; 23 March 2025) have registered 1,453 specific and infraspecific names in Lycoperdaceae, but the actual number of species has been estimated to be around 430 (He et al. 2024; Hyde et al. 2024).

Historically, the genera of Lycoperdaceae were classified by morphological characteristics such as the presence of capillitium or paracapillitium, the type of capillitium (*Lycoperdon*-type, *Bovista*-type, *Calvatia*-type, intermediate *Bovista*-*Lycoperdon* type, and *Mycenastrum*-type), the apical opening type of endoperidium, and the presence or absence of the pseudostipe (Kreisel 1969; Pegler et al. 1995; Larsson and Jeppson 2008).

*Lycoperdon* is the type genus of this family, Lycoperdaceae, and Larsson and Jeppson (2008) recommended a wide-sense concept of *Lycoperdon* based on molecular phylogenetics, in which this family incorporates some traditional genera such as *Bovistella*, *Morganella*, and *Vascellum*. However, some recent studies showed the taxonomic arrangement of five subgenera (*Bovistella*, *Lycoperdon*, *Morganella*, *Utraria* Quél., and *Vascellum*) did not fit well with some taxa (Bates et al. 2009; Gube and Piepenbring 2009; Jeppson and Larsson 2010; Alfredo et al. 2017).

The delineation of genera within the family Lycoperdaceae has undergone significant changes based on the integration of morphological characteristics (Kreisel 1969; Hibbett et al. 1997; Krüger et al. 2001; Moncalvo et al. 2002; Krüger and Kreisel 2003; Bates 2004; Vizzini and Ercole 2017; Krakhmalnyi et al. 2023). With the contributions of numerous prominent mycologists, the taxonomic system of Lycoperdaceae was converging toward a comprehensive understanding of its natural divergence history. Based on ITS and nLSU sequences, the first molecular phylogenetic analysis of Lycoperdaceae was conducted (Larsson and Jeppson 2008), which includes *Lycoperdon*, *Bovista*, *Calvatia*, and *Disciseda* (Krüger et al. 2001; Larsson and Jeppson 2008; Bates et al. 2009; Larsson et al. 2009; Alfredo et al. 2017). A comprehensive phylogeny of the family Lycoperdaceae was reconstructed using sequences from ITS+nLSU+*rpb2*+TEF1-α genetic markers, in which divergence times were estimated for taxa, serving as an additional criterion for taxonomic classification. Combined with morphological analyses, the updated phylogenetic framework supported the division of Lycoperdaceae into 19 genera with divergence times spanning 26.7–75.5 million years ago (Li et al. 2024).

During investigations on puffball fungi in China, we collected many puffball fungi specimens. The objectives of the present study are to explore new fungal taxa in Lycoperdaceae and to reveal the phylogenetic relationships of puffball fungi. Based on the morphological and molecular phylogenetic study, we discovered one new genus, *Lycoperdia*, and three new species, *Calvatiaphlebioides*, *Lycoperdiatomentosa*, and *Morganellaminima*, on the basis of ITS, nLSU, and *rpb2* sequences.

## ﻿Materials and methods

### ﻿Sample collection and examination

The fresh basidiomes of fungi growing on the ground were collected from Qingyuan in Guangdong Province, Dehong, and Zhaotong in Yunnan Province, P.R. China. The samples were photographed using a Xiaomi 12 *in situ*, and fresh macroscopic details, such as the color of the basidiomes, the type of exoperidium, and the shape of the basidiomes, were recorded. All the photographs were focus-stacked and merged using Helicon Focus Pro 7.7.5 software. Specimens were dried in an electric food dehydrator at 40 °C (Hu et al. 2022; Zhao et al. 2023a; Dong et al. 2024a, b) and then sealed and stored in an envelope bag and deposited in the herbarium of the Southwest Forestry University (SWFC), Kunming, Yunnan Province, P.R. China.

### ﻿Morphology

The macromorphological descriptions were based on field notes and photos captured in the field and laboratory and followed the color terminology of Petersen (1996). Micromorphological data were obtained from the dried specimens following observation under a light microscope (Dong et al. 2024a; Yang et al. 2025). Drawings were made using a fungus plotter (Zhao et al. 2023a). The measurements and drawings were made from slide preparations stained with Cotton Blue (0.1 mg aniline blue dissolved in 60 g pure lactic acid) and 5% potassium hydroxide. Spore size data, excluding 5% of the measurements from each end of the range, are shown in parentheses. The following abbreviations were used: KOH = 5% potassium hydroxide water solution, CB+ = cyanophilous, CB = cotton clue, CB– = acyanophilous, Q = variation in the L/W ratios between the specimens studied and n = a/b (number of spores (a) measured from a given number (b) of specimens), Q_m_ represented the average Q of basidiospores measured ± standard deviation.

### ﻿DNA extraction, PCR amplification, sequencing, and phylogenetic analyses

The CTAB rapid plant genome extraction kit—DN14 (Aidlab Biotechnologies Co., Ltd., Beijing, China)—was used to obtain genomic DNA from the dried fungal specimens according to the manufacturer’s instructions (Dong et al. 2024a; Yuan et al. 2024; He et al. 2025; Zhang et al. 2025). The extracted DNA was maintained at –20 °C for long-term storage. Three molecular markers were investigated, i.e., internal transcribed spacer (ITS), nuclear large subunit ribosomal RNA (nLSU), and RNA polymerase II subunit 2 (*rpb2*) gene, and the primers and conditions are shown in Table 1. The PCR products were purified and sequenced at Kunming Tsingke Biological Technology Limited Company (Yunnan Province, China). All newly generated sequences were deposited in NCBI GenBank (https://www.ncbi.nlm.nih.gov/genbank/) (Table 2).

**Table 1.** Loci, primers, PCR amplification procedures, and references used in this study.

Name	Abbreviation	Name	Direction	Sequence (5′-3′)	PCR amplification procedures	References
Internal transcribed spacer region of the rDNA	ITS	ITS5	Forward	GGAAGTAAAAGTCGTAACAAGG	94 °C 2 min; 35 cycles of 94 °C 60 s, 55 °C 60 s, 72 °C 2 min; 72 °C 10 min.	White et al. (1990)
		ITS4	Reverse	TCCTCCGCTTATTGATATGC		
Nuclear large subunit ribosomal	nLSU	LR0R	Forward	ACCCGCTGAACTTAAGC	94 °C 2 min; 35 cycles of 94 °C 30 s, 48 °C 1 min, 72 °C 1.5 min; 72 °C 10 min.	Vilgalys and Hester (1990)
		LR7	Reverse	TACTACCACCAAGATCT		
RNA polymerase second largest subunit	*rpb2*	bRPB2-6F	Forward	TGGGGYATGGTNTGYCCYGC	94 °C 2 min; 9 cycles of 94 °C 45 s, 60 °C 45 s, 72 °C 1.5 min; 36 cycles of 94 °C 45 s, 53 °C 1 min, 72 °C 1.5 min; 72 °C 10 min.	Liu et al. (1999)
		bRPB2-7.1R	Reverse	CCCATRGCTTGYTTRCCCAT		

**Table 2.** A list of species, specimens, and GenBank accession numbers of sequences used in this study. [New species are shown in bold; * indicates type material; - indicates data unavailable].

Species Name	Locality	Sample No.	GenBank Accession No.			References
			ITS	nLSU	*rpb2*	
*Abstomaindicum*	UZ-04-19	India	MN231720	–	–	Altaf et al. (2022)
*Abstomapurpureum*	KM162954	England	GQ981488	–	–	Li et al. (2024)
*Apioperdonpyriforme*	QL20170019	China	PP175742	PP175746	–	Li et al. (2024)
*Apioperdonpyriforme*	ZRL20182005	China	PP175743	PP175747	–	Li et al. (2024)
*Bovistacretacea*	ANMH11622	Iceland	DQ112611	–	–	Larsson and Jeppson (2008)
*Bovistacretacea*	MJ5207	Norway	DQ112610	–	–	Larsson and Jeppson (2008)
Bovistacretacea	ANMH11622	Iceland	DQ112611	–	–	Larsson and Jeppson (2008)
*Bovistalitangensis*	HMAS 258800	China	OR792635	OR831301	–	Li et al. (2024)
*Bovistalitangensis*	HMAS 258801	China	OR792636	OR831302	–	Li et al. (2024)
*Bovistanyalamensis*	HMAS 258836	China	OR792637	OR831304	–	Li et al. (2024)
*Bovistanyalamensis*	HMAS 258837	China	OR792638	OR831306	–	Li et al. (2024)
*Bovistaplumbea*	NYGD01	Pakistan	JX183694	–	–	Yousaf et al. (2013)
*Bovistaplumbea*	MJ4856	Sweden	DQ112613	–	–	Larsson and Jeppson (2008)
*Bovistellaemodensis*	HMAS 287485	China	PP175744	PP175752	–	Li et al. (2024)
*Bovistellaemodensis*	HMAS 287486	China	PP175745	PP175753	–	Li et al. (2024)
*Bryoperdonacuminatum*	TO HG191016	Italy	KY581201	KY581199	–	Vizzini and Ercole (2017)
*Bryoperdonacuminatum*	TO HG201016	Italy	KY581202	KY581200	–	Vizzini and Ercole (2017)
*Calvatiabicolor*	LMG756-58	USA	EU833651			Bates et al. (2009)
*Calvatiacandida*	MJ3514	Hungary	DQ112624	–	–	Larsson and Jeppson (2008)
*Calvatiacraniiformis*	Steinke001017	USA	DQ112625	–	–	Larsson and Jeppson (2008)
*Calvatiacyathiformis*	Strain JTT10	USA	MF686508	–	–	Senthilarasu et al. (2018)
*Calvatiacyathiformis*	MP12	Canada	KY706183	–	–	Senthilarasu et al. (2018)
*Calvatiafenzlii*	Strain Jz01	China	FJ772413	–	–	Senthilarasu et al. (2018)
*Calvatiafragilis*	AH 24114	Argentina	AJ486959	–	–	Senthilarasu et al. (2018)
*Calvatiafragilis*	AH 25227	Pakistan	AJ486958	–	–	Senthilarasu et al. (2018)
*Calvatiagigantea*	MJ3566	Sweden	DQ112623	–	–	Larsson and Jeppson (2008)
*Calvatiagigantea*	HMAS 258889	China	OR792628	OR831299	OR853762	Li et al. (2024)
*Calvatiaholothurioides*	LE 287408	Japan	JQ734547	–	–	Rebriev (2013)
*Calvatiaholothurioides*	KA11-0287	South Korea	KJ909662	–	–	Kim et al. (2016)
*Calvatialongisetulosa*	HMAS 258802	Thailand	OR792617	OR831229	OR853757	Li et al. (2024)
*Calvatialongisetulosa*	HMAS 258803	Thailand	OR792618	OR831230	OR853758	Li et al. (2024)
*Calvatianodulata*	BAFC 4549	Argentina	KY366490	–	–	Alfredo et al. (2014)
*Calvatianodulata*	UFRN Fungos 1691	Brazil	KP751206	–	–	Alfredo et al. (2014)
*Calvatiapachydermica*	AN014692	USA	EU833653	–	–	Bates et al. (2009)
***Calvatiaphlebioides***	CL Zhao 33216*	**China**	**PV345681**	**PV345675**	**PV341017**	Present study
***Calvatiaphlebioides***	**CL Zhao 33366**	**China**	**PV345682**	**PV345676**	**PV341018**	Present study
*Calvatiarubroflava*	TFB11269	Argentina	KY559335	–	–	Senthilarasu et al. (2018)
*Calvatiashennongjiaensis*	HMAS 258804	China	OR792621	OR831294	OR853761	Li et al. (2024)
*Calvatiashennongjiaensis*	HMAS 258806	China	OR792623	OR831291	OR853759	Li et al. (2024)
*Calvatiashennongjiaensis*	HMAS 258808	China	OR792625	OR831292	OR853760	Li et al. (2024)
*Calvatiasubbooniana*	HMAS 258809	China	OR792631	OR831296	–	Li et al. (2024)
*Calvatiasubbooniana*	HMAS 258810	China	OR792630	OR831297	–	Li et al. (2024)
*Discisedabovista*	MJ5078	Sweden	DQ112627	–	–	Larsson and Jeppson (2008)
*Discisedacandida*	STB304	USA	EU833654	–	–	Bates et al. (2009)
*Discisedacervina*	FK17016	Argentina	MN338568	–	–	Li et al. (2024)
*Fuscospinanigrescens*	MJ5376	Sweden	DQ112577	–	–	Larsson and Jeppson (2008)
*Fuscospinanigrescens*	HMAS 258881	China	OR792702	OR831256	–	Li et al. (2024)
*Fuscospinascabricapillitia*	HMAS 258812 T	China	OR792678	PP175748	–	Li et al. (2024)
*Globariaaestivalis*	MJ1122	Sweden	DQ112620	–	–	Larsson and Jeppson (2008)
*Globariagyirongensis*	HMAS 258813	China	OR792660	OR831288	–	Li et al. (2024)
*Globariagyirongensis*	HMAS 258814	China	OR792661	OR831287	–	Li et al. (2024)
*Globariajingningensis*	HMAS 258816	China	OR792645	OR831272	–	Li et al. (2024)
*Globariajingningensis*	HMAS 258817	China	OR792642	OR831271	–	Li et al. (2024)
*Globariatestacea*	HMAS 258819	China	OR792656	OR831280	–	Li et al. (2024)
*Holocotylonbiconicum*	HMAS 258778	China	OR792667	OR831266		Li et al. (2024)
*Holocotylondermoxanthum*	MJ4856	Sweden	DQ112579	–	–	Larsson and Jeppson (2008)
*Holocotylonrupicola*	MJ4304	Norway	DQ112580	–	–	Jeppson et al. (2012)
*Holocotylonrupicola*	MJ7007	Sweden	JN572902	–	–	Jeppson et al. (2012)
*Leptocaulisericaeus*	MJ5395	Sweden	DQ112605	–	–	Larsson and Jeppson (2008)
*Leptocaulisericaeus*	KA13-1463	South Korea	KP340185	–	–	Kim et al. (2016)
*Leptocaulusalbiperidia*	KA12-1210	South Korea	KP340182	–	–	Kim et al. (2016)
*Leptocaulusalbiperidia*	KA121551 t	South Korea	KP340183	–	–	Kim et al. (2016)
*Leptocaulusmuscorum*	MJ9017	Sweden	JN572905	–	–	Jeppson et al. (2012)
*Leptocaulussublongistipes*	HMAS 258775	China	OR792741	OR831318	–	Li et al. (2024)
*Leptocaulussubumbrinus*	HMAS 258885	China	OR792673	OR831315	–	Li et al. (2024)
*Leptocaulussubumbrinus*	HMAS 258886	China	OR792675	OR831317	–	Li et al. (2024)
***Lycoperdiatomentosa***	CL Zhao 37502*	**China**	**PV345683**	**PV345677**	–	**Present study**
***Lycoperdiatomentosa***	**CL Zhao 45073**	**China**	**PV345684**	**PV345678**	–	**Present study**
*Lycoperdiscuslividus*	MJ4005	Sweden	DQ112600	–	–	Larsson and Jeppson (2008)
*Lycoperdiscuslividus*	Dobremez 19740514	Nepal	DQ112599	–	–	Larsson and Jeppson (2008)
*Lycoperdiscustianzhuensis*	HMAS 258767	China	OR792725	OR831324	–	Li et al. (2024)
*Lycoperdiscustianzhuensis*	HMAS 258766	China	OR792725	OR831324	–	Li et al. (2024)
*Lycoperdonnorvegicum*	MJ5453	Sweden	DQ112631	–	–	Larsson and Jeppson (2008)
*Lycoperdonperlatum*	HMAS 258865	China	OR792758	OR831262	–	Li et al. (2024)
*Lycoperdonperlatum*	HMAS 258866	China	OR792763	OR831257	–	Li et al. (2024)
*Lycoperdonsubperlatum*	HMAS 258873	China	OR792751	OR831261	–	Li et al. (2024)
*Lycoperdonsubperlatum*	HMAS 258875	China	OR792750	OR831260	–	Li et al. (2024)
*Morganellaalbostipitata*	INPA239563	Brazil	KU958363	KU958364	–	Alfredo et al. (2017)
*Morganellaalbostipitata*	UFRN-Fungos2249	Brazil	KU958361	KU958362	–	Alfredo et al. (2017)
*Morganellaalbostipitata*	UFRN-Fungos2569	Brazil	KU958357	KU958358	–	Alfredo et al. (2017)
*Morganellaalbostipitata*	UFRN-Fungos2572	Brazil	KU958359	KU958360	–	Alfredo et al. (2017)
*Morganellafuliginea*	UFRN-Fungos2582	Brazil	KU958339	KU958340	–	Alfredo et al. (2017)
*Morganellafuliginea*	UFRN-Fungos2586	Brazil	KU958343	KU958344	–	Alfredo et al. (2017)
*Morganellafuliginea*	UFRN-Fungos2579	Brazil	KU958349	KU958350	–	Alfredo et al. (2017)
***Morganellaminima***	CL Zhao 40537*	**China**	**PV345685**	**PV345679**	–	**Present study**
***Morganellaminima***	**CL Zhao 45072**	**China**	**PV345686**	**PV345680**	–	**Present study**
*Morganellanuda*	UFRN-Fungos2568	Brazil	KU958313	KU958314	–	Alfredo et al. (2017)
*Morganellanuda*	UFRN-Fungos1766	Brazil	KU958315	KU958316	–	Alfredo et al. (2017)
*Morganellanuda*	UFRN-Fungos 2565	Brazil	KU958311	KU958312	–	Alfredo et al. (2017)
*Morganellanuda*	ICN 154541	Brazil	KU958317	KU958318	–	Alfredo et al. (2017)
*Morganellanuda*	UFRN-Fungos 1765	Brazil	KU958319	KU958320	–	Alfredo et al. (2017)
*Morganellaoblongata*	UFRN-Fungos2570	Brazil	KU958355	KU958356	–	Alfredo et al. (2017)
*Morganellapurpurascens*	MEL 2382736	Australia	KP012918	–	–	Alfredo et al. (2017)
*Morganellasosinii*	YR2013	Russia	KC591769	–	–	Alfredo et al. (2017)
*Morganellasubincarnata*	REG106	Germany	AJ237626	–	–	Alfredo et al. (2017)
*Morganellasubincarnata*	TNS Kasuya B286	Japan	KF551244	–	–	Alfredo et al. (2017)
*Morganellatricolor*	HMAS 287487	China	PP175741	PP175750	–	Li et al. (2024)
*Mycenastrumcorium*	MJ5467	Sweden	DQ112628	–	–	Larsson and Jeppson (2008)
*Pseudoperdonmedogense*	HMAS 258784	China	OR792745	OR831254	–	Li et al. (2024)
*Pseudoperdonmedogense*	HMAS 258785	China	OR792746	OR831255	–	Li et al. (2024)
*Pseudoperdonsubcretaceum*	MJ9032	Sweden	JN572908	–	–	Jeppson et al. (2012)
*Sinoperdoncaudatum*	RGC920818	Sweden	DQ112633	–	–	Jeppson and Larsson (2010)
*Sinoperdongyirongense*	HMAS 258787	China	OR792686	OR831248	–	Li et al. (2024)
*Sinoperdongyirongense*	HMAS 258788	China	OR792699	OR831243	–	Li et al. (2024)
*Utrariaexcipuliformis*	HMAS 258850	China	OR792705	OR831326	–	Li et al. (2024)
*Utrariaexcipuliformis*	HMAS 258851	China	OR792703	OR831328	–	Li et al. (2024)
*Vascellumcurtisii*	HMAS 258878	China	OR792665	OR831258	–	Li et al. (2024)
*Vascellumcurtisii*	HMAS 258879	China	OR792666	OR831259	–	Li et al. (2024)
*Vascellumintermedium*	STB091	USA	EU833667	–	–	Bates et al. 2009
*Vascellumpratense*	MJ5858	Czechia	DQ112556	–	–	Larsson and Jeppson (2008)
*Vascellumpratense*	HMAS 258880	China	OR792664	PP175749	–	Li et al. (2024)

**Figure 1.**
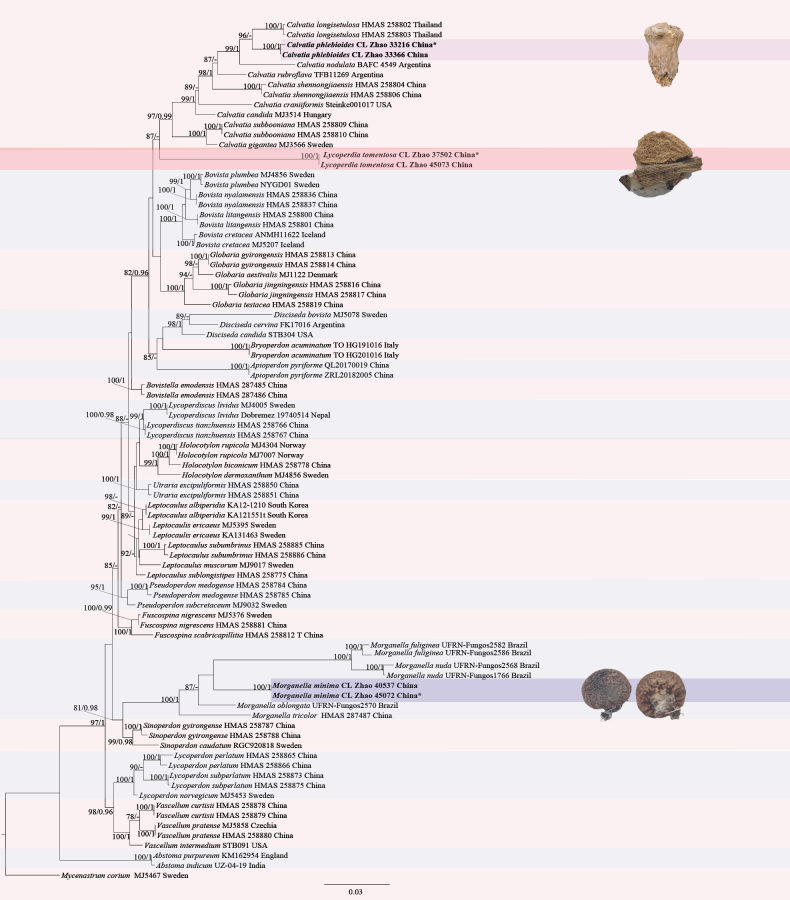
Maximum likelihood strict consensus tree illustrating the phylogeny of one new genus and three new species and related species in Lycoperdaceae based on ITS+nLSU sequences. Branches are labeled with maximum likelihood bootstrap values ≥ 70% and Bayesian posterior probabilities ≥ 0.95, respectively. New species are in bold, * type material.

**Figure 2.**
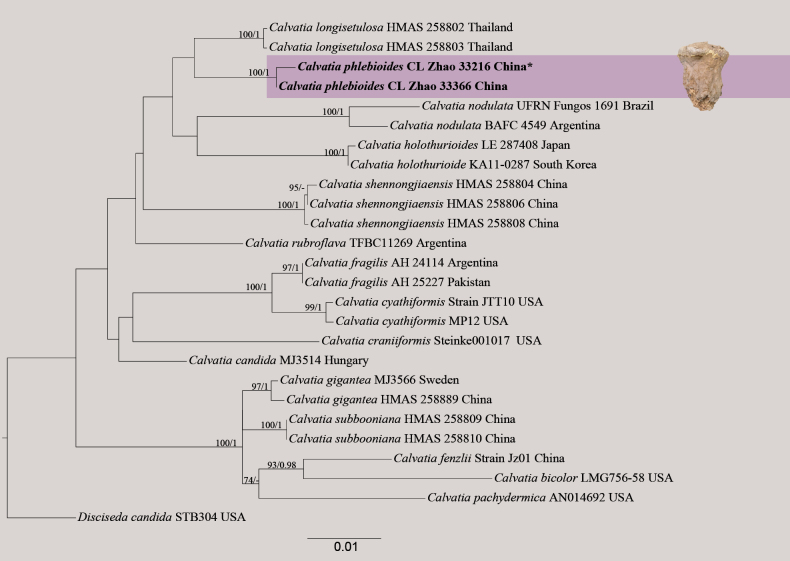
Maximum likelihood strict consensus tree illustrating the phylogeny of *Calvatiaphlebioides* and related species in *Calvatia* based on ITS+nLSU+*rpb2* sequences. Branches are labeled with maximum likelihood bootstrap values ≥ 70% and Bayesian posterior probabilities ≥ 0.95, respectively. New species accessions are in bold, * type material.

**Figure 3.**
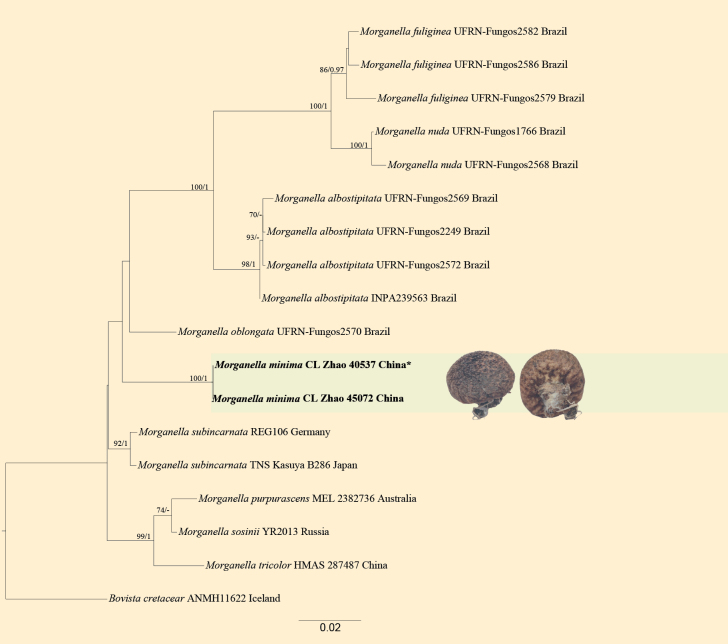
Maximum likelihood strict consensus tree illustrating the phylogeny of *Morganellaminima* and related species in *Morganella* based on ITS+nLSU sequences. Branches are labeled with maximum likelihood bootstrap values ≥ 70% and Bayesian posterior probabilities ≥ 0.95, respectively. New species accessions are in bold, * type material.

Sequences generated for this study were aligned with additional sequences downloaded from GenBank. Sequences were aligned in MAFFT 7 (https://mafft.cbrc.jp/alignment/server/), adjusting the direction of nucleotide sequences according to the first sequence (accurate enough for most cases) and selecting the G-INS-i iterative refinement method (Katoh et al. 2019). The alignment was adjusted manually using AliView version 1.27 (Larsson 2014). The dataset was aligned first, and then the sequences of ITS+nLSU+*rpb2* were combined with Mesquite v. 3.51. The combined ITS+nLSU+*rpb2* sequences and ITS+nLSU datasets were used to infer the positions of the new species and related species. The sequence of *Mycenastrumcorium* (Guers.) Desv. was retrieved from GenBank and used as outgroup taxa in the ITS+nLSU analysis (Fig. 1) in the family Lycoperdaceae (Li et al. 2024); *Discisedacandida* (Schwein.) Lloyd was selected as the outgroup taxon for the ITS+nLSU+ *rpb2* analysis (Fig. 2) in the genus *Calvatia* (Li et al. 2024); *Bovistacretacea* T.C.E. Fr. was selected as the outgroup taxon for the ITS+nLSU analysis (Fig. 3) in the genus *Morganella* (Li et al. 2024).

Maximum likelihood (ML) and Bayesian inference (BI) analyses were applied to the combined two datasets following a previous study (Zhao and Wu 2017; Xu et al. 2025). Maximum likelihood (ML) analysis was performed using the CIPRES Science Gateway (Miller et al. 2012) based on the dataset using the RAxML-HPC BlackBox tool, with RAxML-HPC BlackBox halted after bootstrapping automatically 0.25 with maximum hours and obtaining the best tree using ML search. Other parameters in ML analysis used default settings, and statistical support values were obtained using nonparametric bootstrapping with 1,000 replicates.

The best evolutionary model of each alignment was estimated using jModelTest (Guindon and Gascuel 2003; Posada 2008) under the Akaike information criterion. MrModeltest 2.3 (Nylander 2004) was used to determine the best-fit evolution model for the dataset for Bayesian inference (BI). Bayesian inference was performed with MrBayes 3.1.2 with a general time reversible (GTR+I+G) model of DNA substitution and a gamma distribution rate variation across sites (Ronquist et al. 2012). Branches were considered significantly supported if they received a maximum likelihood bootstrap value (BS) of ≥ 70% or Bayesian posterior probabilities (BPP) of ≥ 0.95.

## ﻿Results

### ﻿Phylogenetic analyses

The combined ITS+nLSU dataset (Fig. 1) included sequences from 86 fungal specimens representing 54 species. The best model for the ITS+nLSU dataset estimated and applied in the Bayesian analysis was GTR+I+G (lset nst = 6; rates = invgamma; prset statefreqpr = dirichlet (1, 1, 1, 1). The Bayesian and ML analyses resulted in a similar topology to that of the MP analysis, with an average standard deviation of split frequencies = 0.009598 (BI), and the effective sample size (ESS) across the two runs is double the average ESS (avg. ESS) = 558. Branches that received bootstrap support for ML and BI ≥ 70% and 0.95 were considered significantly supported, respectively. The results of BLAST queries in NCBI, based on ITS+nLSU separately, showed the sequences producing significant alignment descriptions.

The combined ITS+nLSU+*rpb2* dataset (Fig. 2) included sequences from 26 fungal specimens representing 16 species. The best model for the ITS+nLSU+*rpb2* dataset estimated and applied in the Bayesian analysis was GTR+I+G (lset nst = 6; rates = invgamma; prset statefreqpr = dirichlet (1, 1, 1, 1). The Bayesian and ML analyses resulted in a similar topology to that of the MP analysis, with an average standard deviation of split frequencies = 0.006367 (BI), and the effective sample size (ESS) across the two runs is double the average ESS (avg. ESS) = 1,441. Branches that received bootstrap support for ML and BI ≥ 70% and 0.95 were considered significantly supported, respectively. The results of BLAST queries in NCBI, based on ITS+nLSU+*rpb2* separately, showed the sequences producing significant alignment descriptions.

The combined ITS+nLSU dataset (Fig. 3) included sequences from 18 fungal specimens representing 10 species. The best model for the ITS+nLSU dataset estimated and applied in the Bayesian analysis was GTR+I+G (lset nst = 6; rates = invgamma; prset statefreqpr = dirichlet (1, 1, 1, 1). The Bayesian and ML analyses resulted in a similar topology to that of the MP analysis with an average standard deviation of split frequencies = 0.008915 (BI), and the effective sample size (ESS) across the two runs is double the average ESS (avg. ESS) = 1243.5. Branches that received bootstrap support for ML and BI ≥ 70% and 0.95 were considered significantly supported, respectively. The results of BLAST queries in NCBI, based on ITS+nLSU separately, showed the sequences producing significant alignment descriptions.

In the ITS BLAST results of *Calvatiaphlebioides*, the top ten taxa were *C.holothurioides* Rebriev and *C.candida* (Rostk.) Hollós (maximum record descriptions: max score 1096; total score 1096; query cover 98%; E value 0; ident 94.63%). In nLSU BLAST results, the top ten taxa were *Bovistanigrescens* Pers., *Lycoperdonperlatum*, *Calvatiacandida*, *C.craniiformis*, and *C.caatingaensis* R.L. Oliveira, R.J. Ferreira, B.D.B. Silva, M.P. Martín & Baseia (Maximum record descriptions: Max score 1648; Total score 1648; Query cover 100%; E value 0.0; Ident 97.81%). In *rpb2* BLAST results, the top ten taxa were *Calvatialongisetulosa* R.L. Zhao & J.X. Li, *C.shennongjiaensis* R.L. Zhao & J.X. Li, *C.rubroflava* (Cragin) Lloyd, *Globariamuscicola* R.L. Zhao & J.X. Li, *Bovistellaemodensis* R.L. Zhao & J.X. Li, and *Bovistaplumbea* Pers. (Maximum record descriptions: Max score 966; Total score 966; Query cover 88%; E value 0.0; Ident 91.91%).

In the ITS BLAST results of *Lycoperdiatomentosa*, the top ten taxa were *Morganellapuiggarii* (Speg.) Kreisel & Dring (maximum record descriptions: max score 771; total score 771; query cover 93%; E value 0; ident 89.82%). In nLSU BLAST results, the top ten taxa were *Bovistanigrescens* Pers. and *Lycoperdonperlatum* (maximum record descriptions: max score 1517; total score 1517; query cover 98%; E value 0.0; ident 94.76%).

In the ITS BLAST results of *Morganellaminima*, the top ten taxa were *Morganellasubincarnata* (Peck) Kreisel & Dring, *Lycoperdonpurpurascens* Berk. & M.A. Curtis, and *M.fuliginea* (Berk. & M.A. Curtis) Kreisel & Dring (maximum record descriptions: max score 1026; total score 1026; query cover 93%; E value 0; ident 94.01%). In nLSU BLAST results, the top ten taxa were *Lycoperdonperlatum* and *Bovistanigrescens* (maximum record descriptions: max score 1690; total score 1690; query cover 99%; E value 0.0; ident 98.64%).

The topology based on ITS+nLSU sequences (Fig. 1) showed that all four new taxa were clustered into the family Lycoperdaceae, in which *Lycoperdiatomentosa* formed a monophyletic lineage. The topology based on ITS+nLSU+*rpb2* sequences (Fig. 2) showed that one new taxon, *Calvatiaphlebioides*, was clustered into the genus *Calvatia*, which was sister to *C.longisetulosa*. The topology based on ITS+nLSU sequences (Fig. 3) showed that *Morganellaminima* was clustered into the genus *Morganella*, forming a monophyletic lineage.

### ﻿Taxonomy

#### ﻿ Calvatia phlebioides

Taxon classificationFungiAgaricalesLycoperdaceae

X. Yang & C.L. Zhao sp. nov.

924A015C-AC60-544C-9177-341EC862630F

858401

Figs 4 , 5

##### Holotype.

China • Yunnan Province, Zhaotong, Yiliang County, Wumengshan National Nature Reserve, 27°47'19.69"N, 104°15'4.78"E, elev. 2300 m, on the ground, 19 September 2023, CLZhao 33216 (SWFC).

**Figure 4.**
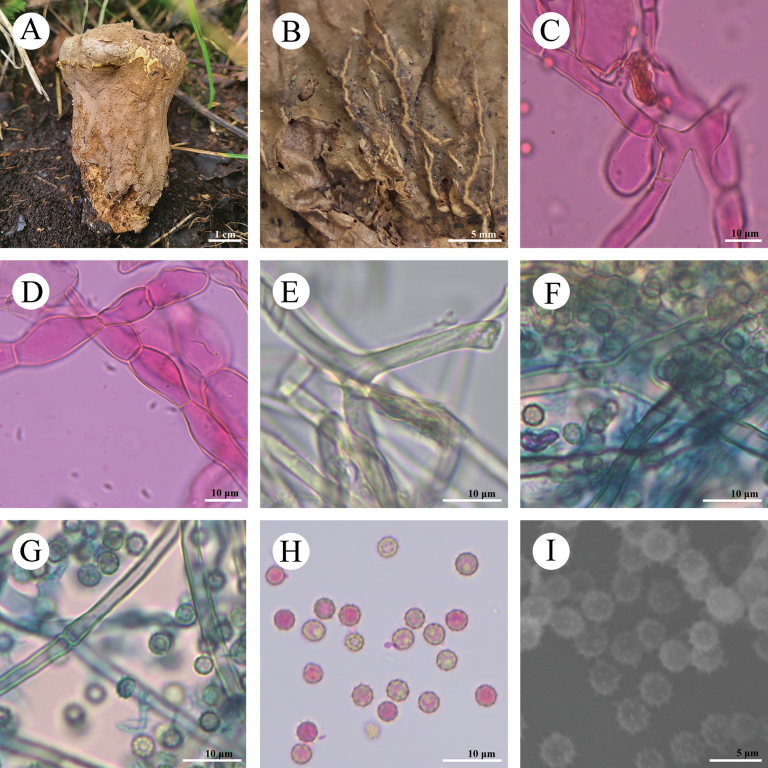
*Calvatiaphlebioides* (CLZhao 33216) **A, B** basidiomes of *C.phlebioides***C, D** exoperidial elements **E** endoperidial hyphae **F, G** capillitial **H** basidiospores **I** basidiospores by SEM.

##### Etymology.

*phlebioides* (Lat.) refers to basidiomes that have a phlebioid surface of the stem base.

**Figure 5.**
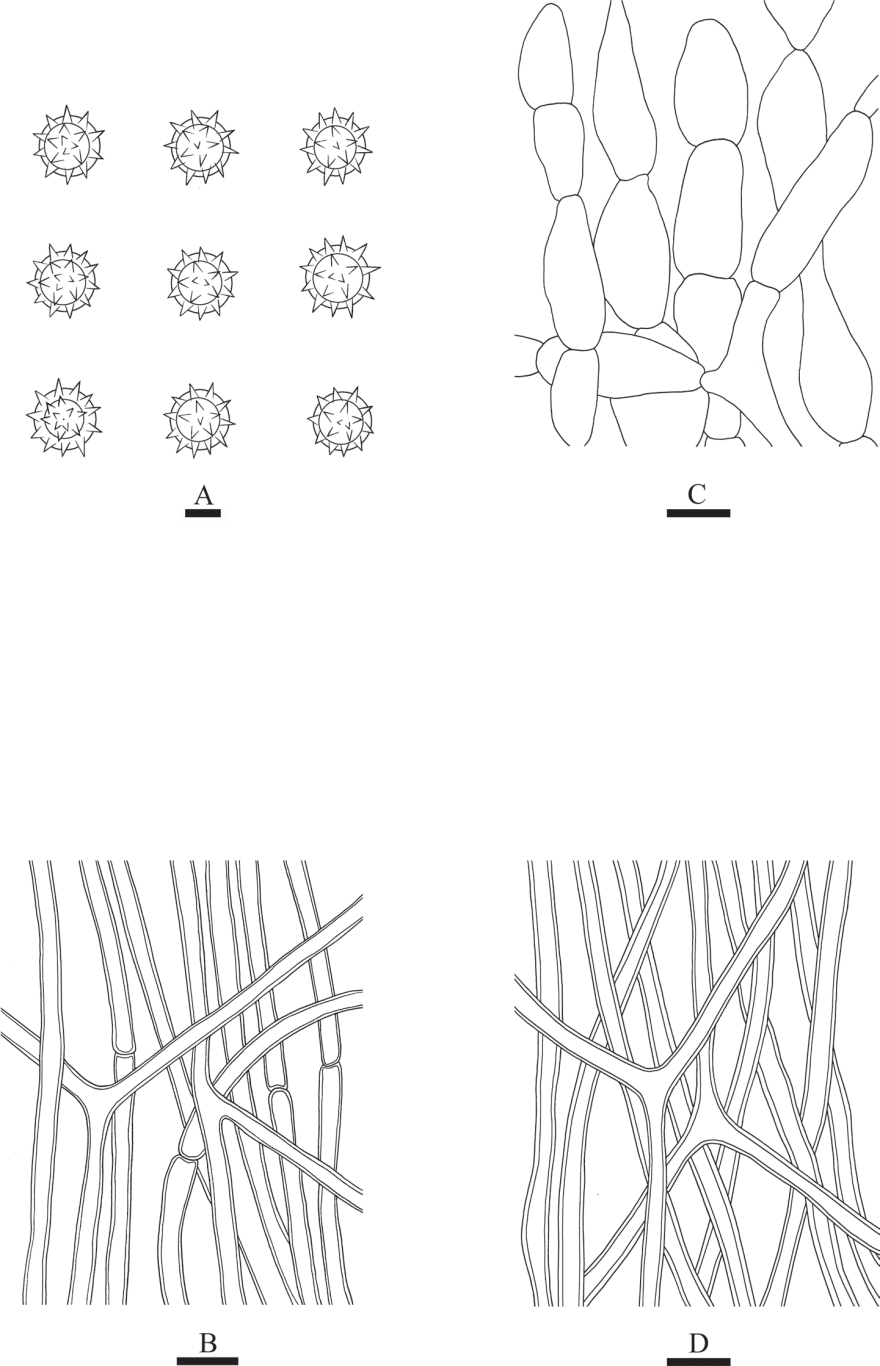
*Calvatiaphlebioides* (CLZhao 33216) **A** basidiospores **B** capillitial **C** exoperidial elements **D** endoperidial hyphae. Scale bars: 1 µm (**A**); 10 µm (**B–D**).

##### Description.

**Fruiting body: *Basidiomes*** broadly obpyriform to turbinate, 30–50 mm in diameter, 45–65 mm in height, slightly tapering at the base, and developing a round base. ***Peridium*** layered: ***Exoperidium*** brownish to dark brownish when mature and grayish-brown when dry, breaking up into thin, irregular-shaped, flaky patches, and the upper part completely detached, the gleba fully exposed. ***Endoperidium*** fragile, bluish-gray when fresh, ash-gray to bluish-gray when dry. ***Gleba*** pale brown to olive-yellow when mature, cottony.

**Hyphal structure: *Exoperidium*** composed of chains of colorless hyphae, ellipsoid, oblong, pyriform, occasionally globose to subglobose, sphaecysts, thin-walled, smooth, colorless in 5% KOH, (16–)20–33(–35) × 10–15 µm. ***Endoperidium*** composed of undulate, colorless hyphae, thick-walled, occasionally branched, 2.5–3.5 µm in diameter. ***Capillitium*** of *Calvatia*-type, rare branched, septate, thick-walled, 2.5–3.6 µm in diameter. *Paracapillitium* absent.

**Basidiospores**: Globose to subglobose, 2.8–3.5(–3.8) × 2.7–3.3(–3.7) μm, honey-yellow, thick-walled, CB–, spinose (less than 1 µm), with a short pedicel (less than 0.5 µm), Q = 1.01–1.02, Q_m_ = 1.02 ± 0.02, n = 60/2.

##### Additional specimen examined (paratype).

China • Yunnan Province, Zhaotong, Yiliang County, Wumengshan National Nature Reserve, 27°47'19.69"N, 104°15'4.78"E, elev. 2300 m, on the ground, 20 September 2023, CLZhao 33366 (SWFC).

#### ﻿ Lycoperdia

Taxon classificationFungiAgaricalesLycoperdaceae

X. Yang & C.L. Zhao gen. nov.

880A9AE9-F053-57E7-8567-070FC3BFA121

858402

##### Type species.

*Lycoperdiatomentosa* X. Yang & C.L. Zhao

##### Etymology.

*Lycoperdia* (Lat.) refers to the new genus resembling *Lycoperdon* in basidiome morphology.

##### Description.

*Basidiomes* pyriform and subglobose to globose when dry. *Exoperidium* with densely arranged tomentose structures. *Endoperidium* papery and fragile. *Gleba* with cottony texture when dry. *Exoperidium* made up of chains of inflated cells, ellipsoid, oblong, pyriform, thin-walled to slightly thick-walled, smooth, colorless hyphae in 5% KOH. *Endoperidium* made up of colorless hyphae, thick-walled, occasionally branched, no septa. *Capillitium* of *Lycoperdon*-type, branched, thick-walled, no septa. *Paracapillitium* composed of chains of colorless, inflated cells, branched, no septa. *Basidiospores* globose to subglobose, with distinct spines, with a short pedicel.

##### Notes.

In our phylogenetic analyses (Fig. 1), *Lycoperdia* is identified as a monophyletic group, typified by *L.tomentosa*. The new genus *Lycoperdia* falls within the family Lycoperdaceae (Agaricales) and is closely related to *Calvatia*. However, *Calvatia* is distinguished from *Lycoperdia* by its dehiscence of peridium, which occurs by irregular fragmentation, and *Calvatia*-type capillitium (Larsson and Jeppson 2008; Li et al. 2024).

#### ﻿ Lycoperdia tomentosa

Taxon classificationFungiAgaricalesLycoperdaceae

X. Yang & C.L. Zhao sp. nov.

B9195053-5AF5-5222-92F1-679A4EB7F65E

858403

Figs 6 , 7

##### Holotype.

China • Yunnan Province, Dehong, Yingjiang County, Tongbiguan Provincial Nature Reserve, 24°28'18.86"N, 97°40'5.60"E, elev. 1000 m, on rotten wood, 3 July 2024, CLZhao 37502 (SWFC).

**Figure 6.**
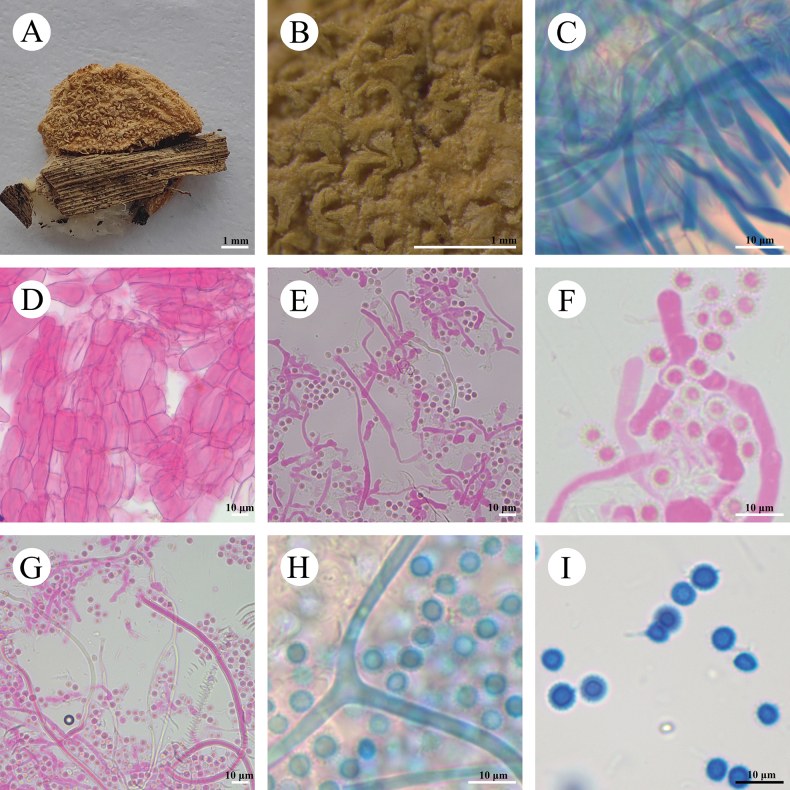
*Lycoperdiatomentosa* (CLZhao 37502) **A, B** basidiomes of *L.tomentosa***C** endoperidial hyphae **D** exoperidial elements **E, F** paracapillitium **G, H** capillitial **I** basidiospores.

##### Etymology.

*tomentosa* (Lat.) refers to the tomentose exoperidium surface.

**Figure 7.**
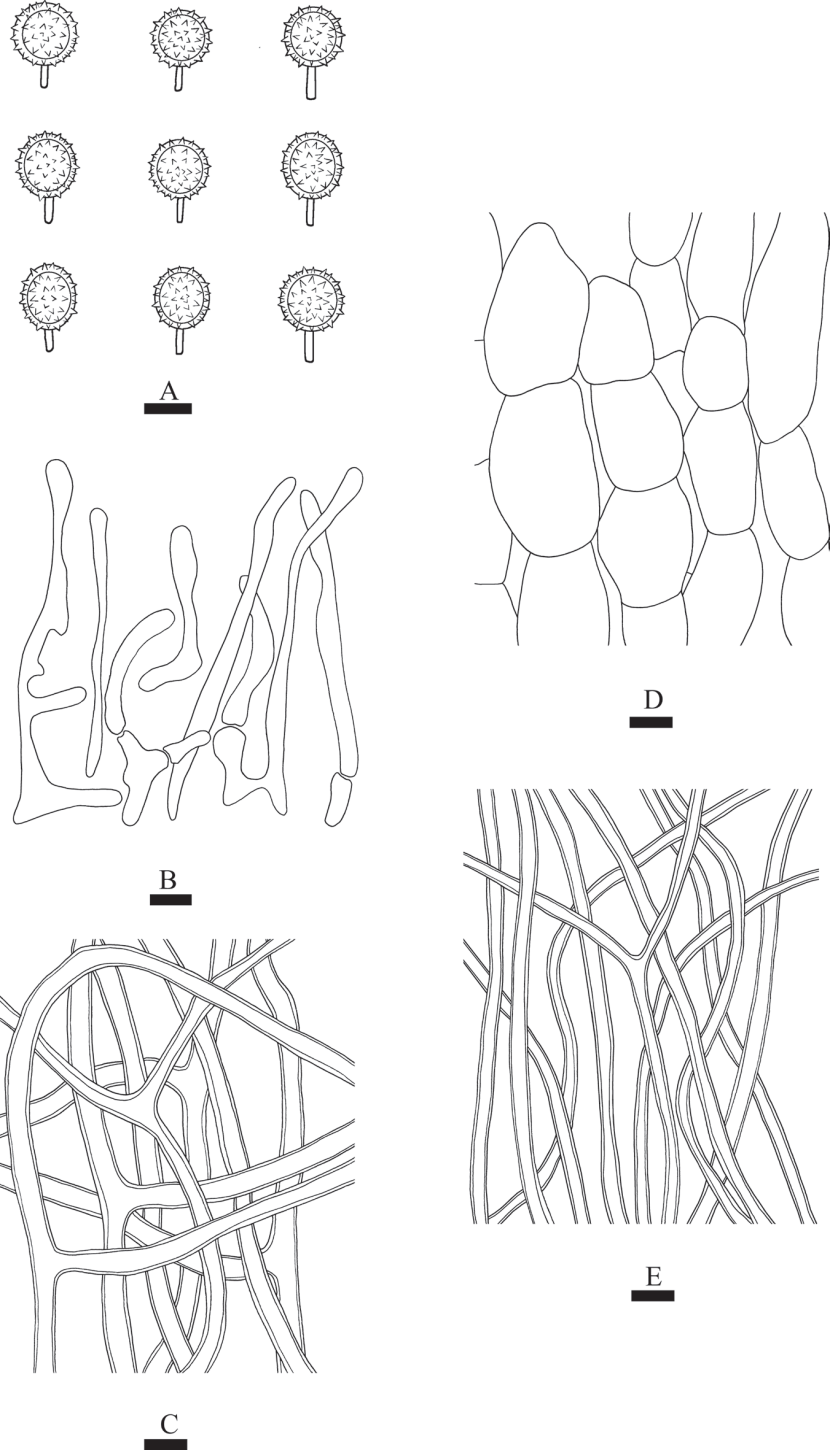
*Lycoperdiatomentosa* (CLZhao 37502) **A** basidiospores **B** paracapillitium **C** capillitial **D** exoperidial elements **E** endoperidial hyphae. Scale bars: 1 µm (**A**); 10 µm (**B–E**).

##### Description.

**Fruiting body: *Basidiomes*** pyriform and subglobose to globose when dry, 5–8 mm in diameter, 6–10 mm in height. ***Peridium*** layered: ***Exoperidium*** sulphur-yellow when fresh, buff-yellow to orange-yellow when dry, with densely arranged tomentose structures, not easily falling off. ***Endoperidium*** fragile, white when fresh, off-white when dry. ***Gleba*** white, powdery, or fibrous.

**Hyphal structure: *Exoperidium*** composed of chains of inflated cells, ellipsoid to oblong, thin-walled to slightly thick-walled, colorless in 5% KOH, 25–40 × 15–25 µm. ***Endoperidium*** made up of colorless hyphae, thick-walled, occasionally branched, no septa, 2.5–4 μm in diameter. ***Capillitium*** of ***Lycoperdon***-type, branched, thick-walled, no septa, 3.5–5 μm in diameter. ***Paracapillitium*** composed of chains of colorless, inflated cells, 2.5–5.5 μm width.

**Basidiospores**: Globose to subglobose, (3.3–)3.5–4.8(–5.3) × (3–)3.2–4.5(–4.8) μm, CB+, ash-gray, thick-walled, with distinct spines, with a long pedicel (1.0–6.5 μm), Q = 1.06–1.07, Q_m_ = 1.06 ± 0.09, n = 60/2.

##### Additional specimen examined (paratype).

China • Yunnan Province, Dehong, Yingjiang County, Tongbiguan Provincial Nature Reserve, 24°28'18.86"N, 97°40'5.60"E, elev. 1000 m, on rotten wood, 18 January 2025, CLZhao 45073 (SWFC).

#### ﻿ Morganella minima

Taxon classificationFungiAgaricalesStrophalosiidae

X. Yang & C.L. Zhao sp. nov.

DFC83A56-9349-54BB-BB24-B55F755A1134

858404

Figs 8 , 9

##### Holotype.

China • Guangdong Province, Qingyuan, Yingde City, Shimentai National Nature Reserve, 24°25'45.17"N, 113°14'58.4"E, elev. 1100 m, on the ground, 15 September 2024, CLZhao 40537 (SWFC).

**Figure 8.**
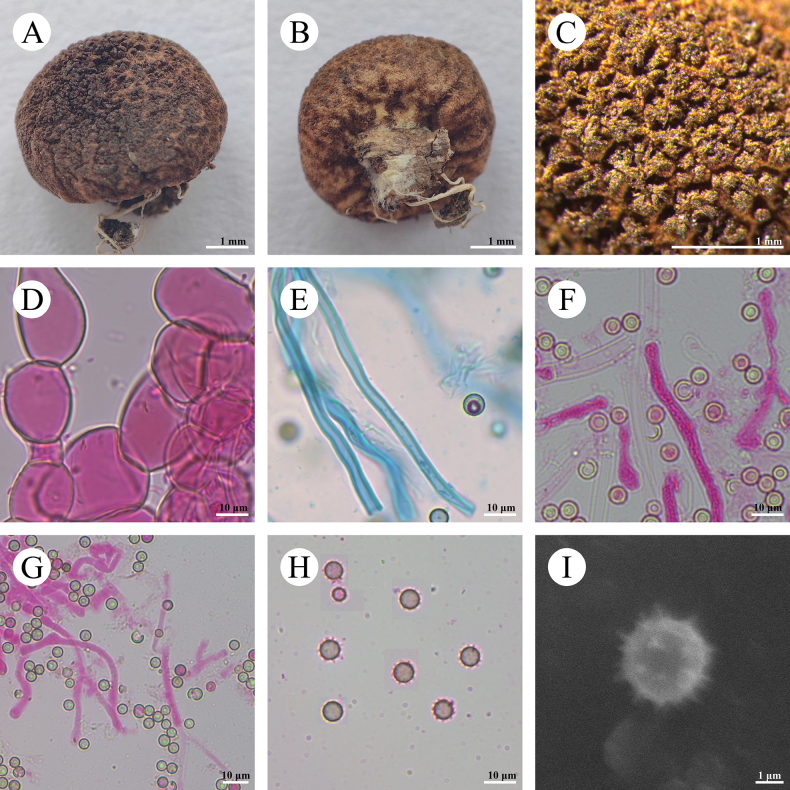
*Morganellaminima* (CLZhao 40537) **A–C** basidiomes of *C.phlebioides***D** exoperidial elements **E** endoperidial hyphae **F, G** paracapillitium **H** basidiospores **I** basidiospores by SEM.

##### Etymology.

*minima* (Lat.) refers to the type species having tiny basidiomes.

**Figure 9.**
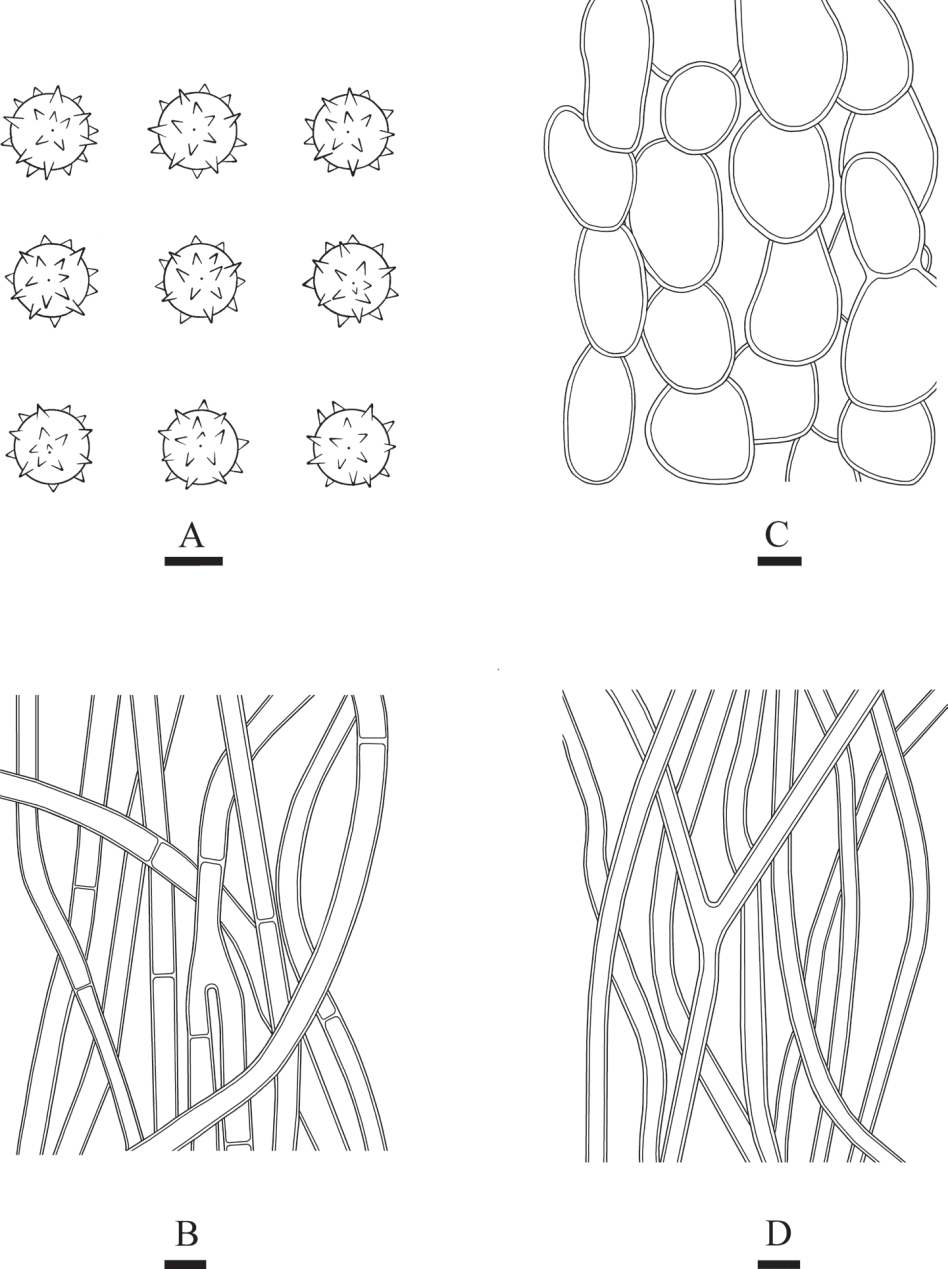
*Morganellaminima* (CLZhao 40537) **A** basidiospores **B** paracapillitium **C** exoperidial elements **D** endoperidial hyphae. Scale bars: 1 µm (**A**); 10 µm (**B–D**).

##### Description.

**Fruiting body: *Basidiomes*** subglobose, 5–8 mm in diameter, 3–7 mm in height. ***Peridium*** layered: ***Exoperidium*** slightly brown when fresh, grayish-brown to fuscous when dry, hymenial surface with coarse granular, not easily falling off, densely grouped on the top of the basidioma; ***Endoperidium*** fragile, cream to slightly brown when fresh, slightly brown upon drying. ***Gleba*** fuscous, powdery.

**Hyphal structure: *Exoperidium*** composed of irregular inflated cells, globose, subglobose, or pyriform, thin-walled, colorless in 5% KOH, 15–50 (–90) × (10) 15–32 (–40) μm. ***Endoperidium*** made up of intertwined colorless hyphae, thick-walled, slightly branched, 3–5(–7.5) μm in diameter. ***Capillitium*** absent. ***Paracapillitium*** interwoven and branched, thick-walled, 4–7 μm in diameter, septate.

**Basidiospores**: Globose to subglobose, 4.7–5.5(–6.3) × 4.6–5.5(–6.2) μm in diameter, brownish, CB–, thin-walled, ornamented with distinct spines (less than 1 µm), with a short pedicel (less than 0.5 µm), Q = 1.02–1.03, Q_m_ = 1.02 ± 0.02, n = 60/2.

##### Additional specimen examined (paratype).

China • Guangdong Province, Qingyuan, Yingde City, Shimentai National Nature Reserve, 24°25'45.17"N, 113°14'58.4"E, elev. 1100 m, on the ground, 17 January 2025, CLZhao 45072 (SWFC).

## ﻿Discussion

In the family Lycoperdaceae, several morphological characteristics act as distinguishing features for its genera, which include the existence and type of capillitium, the presence or lack of paracapillitium, the existence and structure of subgleba, the opening modes of the endoperidium, and the anatomy of the peridium (Larsson and Jeppson 2008; Bates et al. 2009; Larsson et al. 2009; Jeppson and Larsson 2010; Kim et al. 2016; Krakhmalnyi et al. 2023; Li et al. 2024). In the present study, one new genus, *Lycoperdia*, and three new species, *Calvatiaphlebioides*, *Lycoperdiatomentosa*, and *Morganellaminima*, are described based on phylogenetic analyses and morphological characteristics.

Phylogenetically, the phylogram based on the combined ITS+nLSU sequences (Fig. 1) showed that one new genus, *Lycoperdia*, formed a monophyletic clade, and the new species, *Lycoperdiatomentosa*, was assigned to the genus *Lycoperdia* within the family Lycoperdaceae. However, morphologically, *Lycoperdon* differs from *Lycoperdia* in its peridium dehiscing by a definite apical stoma (Cunningham 1926b).

Based on the combined ITS+nLSU+*rpb2* sequences (Fig. 2), the phylogram showed that *Calvatiaphlebioides* was assigned to the genus *Calvatia*, in which *C.phlebioides* was retrieved as a sister to *C.longisetulosa*. However, morphologically the species *C.longisetulosa* differs from *C.phlebioides* by having the exoperidium densely covered with robust spines and longer pedicels present (0.5–1 µm; Li et al. 2024).

Phylogenetically, the phylogram based on the combined ITS+nLSU sequences (Fig. 3) showed that *Morganellaminima* was assigned to the genus *Morganella* and formed a monophyletic lineage with *M.oblongata* (Accioly, Baseia & M.P. Martín) R.L. Zhao & J.X. Li. However, morphologically, *M.oblongata* differs from *M.minima* by having larger basidiomes (10 mm in height, 15.5 mm in diameter; Alfredo et al. 2017).

Morphologically, *Lycoperdia* resemble the other fourteen genera in the family Lycoperdaceae: *Abstoma*, *Apioperdon*, *Bovista*, *Bovistella* Morgan, *Bryoperdon*, *Calbovista* Morse ex M.T. Seidl, *Calvatia*, *Disciseda*, *Fuscospina* R.L. Zhao & J.X. Li, *Gastropila*, *Lycoperdon*, *Morganella*, *Pseudoperdon* R.L. Zhao & J.X. Li, and *Sinoperdon* R.L. Zhao & J.X. Li. A morphological comparison between the new genus *Lycoperdia* and the other fourteen genera is presented in Table 3.

**Table 3.** A morphological comparison between the new genus *Lycoperdia* and fourteen other genera in the family Lycoperdaceae.

Species name	*Basidiomes*	Exoperidium	Endoperidium	Gleba	*Capillitium*	*Paracapillitium*	Basidiospores	References
*Abstoma*	Subglobose, stoma absent.	Fragile; breaking away irregularly.	Fragile; dehisces by irregular rupture.	Firm at maturity.	Calvatia-type, occasionally branched, smooth.	–	Apedicellate, globose.	Cunningham (1926a)
*Apioperdon*	Obovoid to obpyriform.	–	–	–	Lycoperdon-type.	–	Smooth to minutely ornamented.	Kreisel (1962)
*Arachnion*	Small-sized;	–	Disintegrates at maturity	Develops minute peridioles resembling sand grains	Absent or poorly developed.	–	Exhibit reticulate ornamentation.	Cortez et al. (2010)
*Bovista*	–	Persistent spines	–		Bovista-type or Bovista-Lycoperdon type.	–	Ellipsoid, pedicellate	Li et al. (2024)
*Bovistella*	Medium-sized; distinct pseudo-diaphragm	–	–	–	Fragile; more or less abundant pits of irregular outline.	–	Subglobose to slightly ellipsoid	Larsson and Jeppson (2008)
*Bryoperdon*	Small, ovoid, with mycelial cords;	–	–	–	Lycoperdon-type.	–	Smooth to minutely pustulose-verrucose	Vizzini and Ercole (2017)
*Calbovista*	Medium to large; top-shaped	Coriaceous, fall away from top downward at maturity	–	Fragile, dark umber at maturit	Abundant discrete, ochraceous yellow, antler-like.	–	–	Morse (1935)
*Calvatia*	Globose to pyriform, turbinate.	Dehiscence of peridium occurring by irregular fragmentation.	–	Pulverulent to cottony.	Calvatia-type.	–	Smooth to verrucose and echinate.	Li et al. (2024)
*Disciseda*	Globose to globose-depressed.	Covered by a sand case.	–	–	Calvatia-type; commonly wavy.	–	Smooth to verrucose, and shortly pedicellate.	Li et al. (2024)
*Fuscospina*	Lycoperdoid; dark brown appearance	Adorned with conical and curved warts.	Sessile; 6–20 mm in diam; obvious oral margin ring.	–	Lycoperdon-type capillitium with pits.	–	covered with rounded warts.	Li et al. (2024)
*Gastropila*	Almost globose, dehiscing in an irregularly radiate-stellate manner.	Fragile.	Corky-spongy.	–	Smooth threads, sparsely branched, not easily broken, much entangled.	–	Smooth.	Homrich and Wright (1973)
*Lycoperdon*	–	Echinate.	Smooth.	–	–	–	Globose to subglobose, usually ornamented with minute warts.	Li et al. (2024)
*Lycoperdia*	Pyriform, and subglobose to globose when dry.	Densely arranged tomentose structures.	Papery and fragile.	Cottony texture when dry.	Lycoperdon-type, branched, thick-walled, no septa.	Composed of chains of colorless, inflated cells, branched, no septa.	Globose to subglobose, with distinct spines, with a short pedicel.	Present study
*Morganella*	Depressed, globose to pyriform.	–	–	Consists of abundant paracapillitium.	Absent.	Abundant.	Asperulate to echinulate.	Li et al. (2024)
*Pseudoperdon*	Globose to subglobose	–	–	–	Lycoperdon-type, with abundant large irregular pores	–	–	Li et al. (2024)
*Sinoperdon*	Lycoperdoid.	Echinate exoperidium covred with conical spines.	–	–	Lycoperdon-type, thick walls.	Abundant.	Verrucose; long pedicels	Li et al. (2024)

*Calvatiaphlebioides* resembles the other ten species in the genus *Calvatia*: *Calvatiabicolor* (Lév.) Kreisel, *C.candida*, *C.craniiformis*, *C.cyathiformis*, *C.fragilis* (Quél.) Morgan, *C.holothurioides* Rebriev, *C.longisetulosa*, *C.nodulata* Alfredo & Baseia, *C.shennongjiaensis*, and *C.subbooniana*. Table 4 presents a morphological comparison between *Calvatiaphlebioides* and the other ten species.

**Table 4.** A morphological comparison between the new species *Calvatiaphlebioides* and ten other species in the genus *Calvatia*.

Species name	Basidiomes	Exoperidium	Endoperidium	Gleba	*Capillitium*	Basidiospores	References
*Calvatiabicolor*	35–68 mm in height, 41–103 mm in diameter; subglobose to globose.	Thin; membranous and fragile; brownish orange to light brown.	Thin; smooth; reddish blond to brownish orange; dehiscing by irregular rupture of whole peridium.	Lanose; persistent; yellowish-brown at maturity.	2.5–6 μm in diameter; smooth and thick-walled; dichotomously branched; septate, without pores.	4.2–5 μm in diameter; globose; strongly echinate (<1.5 μm) long; shortly pedicellate (<1 μm).	Cortez et al. (2012)
*Calvatiacandida*	15–50 mm in height, 20–60 mm in diameter; subglobose, depressed globose to turbinate.	Thin and papery; glabrous to minutely floccose; grayish orange to orange.	Thin and fragile; felted; grayish orange.	Cottony, rather firm; yellow.	3.2–4.8 μm in diameter; thick-walled; straight to subundulate, with abundant pores.	4.0–4.8 × 4.0–4.8 μm; globose to subglobose; echinate; shortly pedicellate (<1.6 μm).	Bates et al. (2009)
*Calvatiacraniiformis*	60–120 mm in height, 70–150 mm in diameter; obovoid, broadly obpyriform to turbinate.	Thin and papery; glabrous to minutely floccose or sparsely encrusted with particles of dirt; white to off-white at first, becoming dark yellow to yellowish brown.	Thin and fragile; glabrous or felted.	Cottony and firm; white to off-white and solid at first, becoming olive yellow, olive-brown to light brown.	4.8 μm in diameter; thick-walled; straight to subundulate, glabrous with abundant pores, septate.	3.2–4.0 × 3.2–4.0 μm; globose to subglobose; smooth to asperulate.	Bates et al. (2009)
*Calvatiacyathiformis*	80–130 mm in height, 70–130 mm in diameter; obovoid, subglobose, depressed globose, broadly obpyriform to turbinate.	Thin and papery; glabrous to minutely floccose or sparsely encrusted with particles of dirt; white to off-white at first, becoming light brown to brown or remaining white, finally turning violet-brown to dark magenta.	Thin and fragile; felted; violet-brown to dull violet or purplish gray.	Cottony, rather firm; white to off-white and solid at first, becoming violet-brown to dark magenta or grayish magenta.	3.2–6.4 μm in diameter; thick-walled; straight to subundulate, glabrous with abundant pores, septate.	5.6–8.0 × 5.6–8.0 μm; globose; strongly and densely verrucose (<1.0 μm).	Bates et al. (2009)
*Calvatiafragilis*	35–60 mm in height, 40–70 mm in diameter; subglobose, depressed globose to turbinate.	Thin and papery; glabrous to appressed floccose; white to off-white at first, becoming pale yellow or remaining white, finally turning violetbrown as the peridia begin to disintegrate.	Thin and fragile; felted; gray to violet-brown.	Cottony, rather firm; white to off-white and solid at first, becoming grayish magenta to dull violet.	3.2–4 μm in diameter; thick-walled; straight to subundulate, glabrous with abundant pores, septate.	6.4–7.2 × 6.4–7.2 μm; globose; verrucose (<0.8 μm).	Bates et al. (2009)
*Calvatiaholothurioides*	40–50 mm in height, 30–55 mm in diameter; pyriform, turbinate to broadly excipuliform.	Thin, fragile, and tomentose; yellow-orange to fulvous.	olive-brown.	cottony, fulvous.	2–4 μm in diameter; thin-walled; branch, septate.	3.4–4.5 × 2.3–3.1 μm; globose; spines (0.4–0.6 μm high); without Pedicels.	Rebriev (2013)
*Calvatialongisetulosa*	20–35 mm in height, 24–40 mm in diameter; pyriform, tdepressed globose.	densely with robust spines, coniform; yellowish-white, yellowish-brown, dark brown to reddish-brown in upper part.	Papery and fragile; whitish.	pulverulent, cottony; initially whitish, grayish brown, light-brown.	1.4–3.6 μm in diameter; thick-walled; straight, occasionally branched and septa.	3.3–3.7 μm in diameter; globose; distinctly ornamented echinate; short pedicels present (0.5–1 μm).	Li et al. (2024)
*Calvatianodulata*	29–56 mm in height, 24–55 mm in diameter; pyriform to turbinate.	granulose to pilose, not persistent, brown in base becoming olive brown in apex.	papery, surface smooth to wrinkly, plicate; light brown to brown.	initially cottony becoming powdery olive brown at maturity.	2–4 μm in diameter; thick-walled; occasionally branched and septate, rare circular pits.	3.1–4.7 × 2.9–4.6 μm in diameter; subovoid to ampulliformis, punctate, pedicels (0.5–3.7 μm).	Alfredo et al. (2014)
*Calvatiaphlebioides*	45–65 mm in height, 30–50 mm in diameter; broadly obpyriform to turbinate	Brownish to dark brownish when mature, grayish-brown when dry.	Fragile; bluish-gray when fresh, ash-gray to bluish-gray when dry.	Cottony; pale brown to olive-yellow when mature.	2.5–3.6 µm in diameter; rare branched, septate, thick-walled.	2.8–3.5 × 2.7–3.3 μm in diameter; spinose (< 1 µm), with a short pedicel (< 0.5 µm).	Present study
*Calvatiashennongjiaensis*	30 mm in height, 35 mm in diameter; broadly obpyriform to turbinate.	initially whitish, ochraceous to dark brownish when mature.	papery and fragile; pale yellow.	Cottony; initially whitish, pale brown to olive-yellow when mature.	straight, rare branched, septate; fragile.	3.2–4.0 μm in diameter; globose to subglobose; spinose (<2 μm); short pedicel (<3 μm).	Li et al. (2024)
*Calvatiasubbooniana*	30–50 mm in height, 30–80 mm in diameter; globose, depressed globose, subovate.	Thin; reenish-olive or sometimes light reddish-brown.	Papery; whitish.	Pulverulent, yellowish-white to dark olive-brown when mature.	2.4–7.2 μm in diameter; thick-walled; occasionally branched and septate, constricted at the septa.	5.2–6.6 × 3.7–5.1 μm in diameter; ovoid, oblong; smooth; short pedicel (<0.5 μm).	Li et al. (2024)

*Morganellaminima* resembles the other ten species in the genus *Morganella*, *M.afra* Kreisel & Dring, *M.austromontana* C.R. Alves, Cortez & R.M. Silveira, *M.benjaminii* (Rick) Cortez, Calonge & Baseia, *M.fimbriata* Rebriev, *M.mengsongensis* (L. Ye, P.E. Mortimer, & Karunarathna) R.L. Zhao & J.X. Li, *M.nuda* Alfredo & Baseia, *M.oblongata*, *M.rimosa* Baseia & Alfredo, *M.sosinii* Rebriev & Bulakh, *M.tricolor* R.L. Zhao & J.X. Li. Table 5 presents a morphological comparison between the new species, *Morganellaminima*, and the other ten species.

**Table 5.** A morphological comparison between the new species *Morganellaminima* and the other ten species in the genus *Morganella*.

Species name	*Basidiomes*	Exoperidium	Endoperidium	Gleba	*Paracapillitium*	Basidiospores	References
*Morganellaafra*	10 mm in diameter; depressed globose to pyriform.	Minutely granular; fuscous above, becoming lighter below.	Thin; smooth to very minutely areolate; fawn.	Becoming grayish as the spores are shed.	–	3.5–4.5 μm in diameter; globose; minutely asperulate to short-spined.	Kreisel and Dring (1967)
*Morganellaaustromontana*	10 mm in height, 8–20 mm in diameter; subglobose to pyriform.	Spiny, formed of short spines (<1 mm high); light brown when fresh.	Papery; smooth to areolate; white when fresh; yellow gray after dried.	Cottony; brownish gray.	7–10 µm in diameter; infated; with numerous small pores.	4–5 × 3–4 μm in diameter; ovoid to subglobose; spiny under SEM (<2 μm).	Alves et al. (2017)
*Morganellabenjaminii*	5–10 mm in diameter; globose, sessile; grayish orange.	Composed of minute spines.	Smooth; fragile.	–	2–4 µm in diameter; thin-walled; more or less branched, smooth, septate.	2.5–3.5 μm in diameter; globose; verrucose, with a pedicel (<1.5 μm).	Cortez et al. (2017)
*Morganellafimbriata*	10–15 mm in height, 11–16 mm in diameter; turbinate to pyriform.	Dark-brown to cream-brown slender spines (0.5–1.5 mm).	Light grayish to cream-brown.	Cream-brown.	5–7 µm in diameter; rarely bifurcated.	2.9–3.4 μm in diameter; globose; verruculose under the SEM, with a pedicel (5–7 μm).	Rebriev (2023)
*Morganellamengsongensis*	11–22 mm in height, 10–23 mm in diameter; globose or depressed globose.	Minute conical tubercles; grayish white to dark gray.	Thin, papery, smooth to wrinkled with age; white to dark brown.	Cottony and white when young, powdery and light brown when mature.	2.7–3.2 µm in diameter; septate, occasionally branched.	2.8–3.7 μm in diameter; subglobose or globose; echinate, spine (<0.5 μm).	Ye et al. (2022)
*Morganellaminima*	3–7 mm in height, 5–8 mm in diameter; subglobose.	Hymenial surface with coarse granular; slightly brown when fresh, grayish-brown to fuscous when dry	Fragile; cream to slightly brown when fresh, slightly brown upon drying	Fuscous; powdery	4–7 μm in diameter; thick-walled; branched.	4.7–5.5 × 4.6–5.5 μm in diameter; globose to subglobose; ornamented with distinct spines (< 1 µm), with a short pedicel (< 0.5 µm).	Present study
*Morganellanuda*	depressed globose to pyriform.	Ornamentation granulose.	Surface smooth; orange when young to yellowish brown.	Powdery; brown.	–	5.5–7.5 μm in diameter; globose; aculeate in SEM (0.5–1 μm).	Alfredo et al. (2017)
*Morganellaoblongata*	10 mm in height, 15.5 mm in diameter; epigeous, depressed globose to subglobose, slightly umbonate.	Formed by a tomentum organized in tufts sphaerocysts; light yellow to light orange.	Smooth; cream.	Cottony; furfuraceous; grayish orange to brownish orange.	3.2–6.3 µm in diameter; branched.	5.4–6.6 μm in diameter; globose; aculeate (<1 μm).	Alfredo et al. (2017)
*Morganellarimosa*	45–55 mm in height, 50–60 mm in diameter; globose to subglobose.	rimose, granulose; pale yellow.	Smooth; pale yellow.	Reddish yellow.	1.5–2 µm in diameter; septate and branched.	2–3 μm in diameter; globose, subglobose to ovoid; verrucose to equinulate, apiculate.	Alfredo et al. (2012)
*Morganellasosinii*	7–15 mm in height, 7–20 mm in diameter; subglobose, depressed-globose.	Consists of granules and echinulate; dark-brown to blackish.	Papery thin; brown, to dark-brown.	Olive brown.	4–6 µm in diameter; septate and unbranched.	2–3 μm in diameter; globose, subglobose to ovoid; verrucose to equinulate, apiculate.	Rebriev and Bulakh (2015)
*Morganellatricolor*	10–13 mm in diameter; subglobose, depressed-globose.	Surface with coarse granular; yellowish to brownish at the upperpart, deep brownish at apical part.	Smooth, fragile; off-white.	Powdery or fibrous; brown.	3.1–5.7 μm in diameter; straight without branches; septa abundant.	3.2–3.6 μm in diameter; globose to subglobose; densely ornamented coarse verrucae, cylindrical or inverted pyramid.	Li et al. (2024)

Macrofungi are an important part of forest ecosystems, which are mainly composed of most members of Basidiomycota, in which they possess important economic values and ecological functions (Chen et al. 2015; Wu et al. 2022a, 2022b; Dai et al. 2021; Deng and Zhao 2023; Guan et al. 2023; Wang et al. 2023, 2024; Yuan et al. 2023a; Dong et al. 2024a, b, 2025; Luo et al. 2024; Zhao et al. 2024). The family Lycoperdaceae is an extensively studied group of Basidiomycota (Larsson and Jeppson 2008; Krakhmalnyi et al. 2023). However, the diversity of puffball fungi in China is still not well known, especially in the subtropical and tropical areas. Puffball fungi have huge potential as food and medicines, especially in Asia, and their prospects (Dai and Yang 2008; Dai et al. 2010; Cui et al. 2023). In the present study, three new species are introduced from China, further enriching our knowledge of the macrofungal diversity. We anticipate that more undescribed Lycoperdaceae taxa will be discovered throughout China after extensive collection combined with morphological and molecular analyses.

## Supplementary Material

XML Treatment for Calvatia phlebioides

XML Treatment for Lycoperdia

XML Treatment for Lycoperdia tomentosa

XML Treatment for Morganella minima

## ﻿Additional information

### Author ORCIDs

Xin Yang https://orcid.org/0009-0009-7755-0581

Songjing Duan https://orcid.org/0009-0005-3122-0318

Mei Li https://orcid.org/0009-0003-6281-4990

Dongxu Li https://orcid.org/0009-0008-3991-1844

Rongcong Yang https://orcid.org/0009-0006-0646-5265

Shihui Zhang https://orcid.org/0009-0008-3309-4214

Taimin Xu https://orcid.org/0000-0002-5230-4140

Wen Li https://orcid.org/0009-0005-1394-4945

Hongmin Zhou https://orcid.org/0000-0002-0724-5815

Changlin Zhao https://orcid.org/0000-0002-8668-1075

### Data availability

All of the data that support the findings of this study are available in the main text.
